# Medicine Adherence and Associated Factors in Immigrants and Refugees: A Systematic Review

**DOI:** 10.1155/2022/1993066

**Published:** 2022-12-28

**Authors:** Damini Patel, Zahraa Jalal, Ping Guo

**Affiliations:** ^1^School of Pharmacy, University of Birmingham, Edgbaston, Birmingham B152TT, UK; ^2^School of Nursing and Midwifery, University of Birmingham, Edgbaston, Birmingham B152TT, UK

## Abstract

Medicine nonadherence is a major contributing factor to morbidity and mortality. Almost half of the chronically ill patients are nonadherent to their medication. Vulnerable groups like immigrants and refugees are at a higher risk of poor medication adherence. This study aims to determine the rate of medicine adherence and the factors associated with medicine nonadherence in a population of immigrants and refugees. A protocol-led (PROSPERO ID: CRD42021285419) systematic review was conducted by searching PubMed, Medline, Embase, Scopus, CINAHL, and Cochrane Library for studies published between 1st January 2000 and 4th November 2021. PRISMA guidelines were followed. The NIH quality assessment tool and CASP checklist were used to quality assess the papers. Data were searched, screened, and extracted. Extracted data were tabulated for descriptive and narrative analyses. 15 studies were conducted across six countries including participants with various medical conditions. The rate of medicine adherence reported ranged from 10.1% to 74.5%. Higher rates of nonadherence were observed in immigrants and refugees compared to migrant and native groups. Socio-economic factors, including language proficiency, level of education, and financial burden, and patient-related factors involving cultural behaviours and beliefs were common themes for nonadherence among immigrants and refugees. Further research is required to address the effect of nonadherence on clinical outcomes. Studies should focus on using a consistent definition of adherence and the same objective methods to measure rates of adherence to allow for meta-analysis of data and definitive results. Healthcare professionals (HCPs) are recommended to target interventions at improving adherence and reducing modifiable risk factors in immigrants and refugees, thus reducing health disparities among the population.

## 1. Introduction

Medicine adherence refers to the extent to which patients take their medication according to agreed recommendations and instructions from their healthcare professional (HCP) [1]. Reviews conducted worldwide found that approximately 50% of chronically ill patients do not adhere to their prescribed medication, with adherence rates decreasing as more time passes or barriers emerge following an initial written prescription [2]. Higher rates have been reported in developing countries due to a scarcity of medical and educational resources. Nonadherence to medicine is a major cause of morbidity and mortality in patients, particularly in vulnerable groups such as immigrants and refugees. A study on 96 Southeast Asian refugees showed that only 12% of patients presenting with various health conditions were adhering to all their prescribed medication [3]. A cohort study also indicated lower adherence rates to antipsychotics in immigrants compared to native-born Koreans, 10.1% and 14.6%, respectively [4]. Nonadherence can be further categorised into intentional or unintentional; understanding the patients' motivation behind not taking their medication will allow for better support and interventions from HCPs.

Poor adherence to medication can have detrimental effects on clinical outcomes and the quality of life of patients, as well as increase the economic burden of treatment of common diseases [5]. There are many barriers to adherence; poor medication adherence can be caused by patient, prescriber, and or medication factors. Low socio-economic status, poor income, side effects, complex medication regimens, lack of understanding, and low motivation are all examples of factors that contribute to medicine nonadherence. Some factors are more prevalent in particular patient populations. Specific patient age groups, races and ethnicities, living situations, and medical conditions have been associated with poorer adherence to medication. Literature has directly linked race and ethnicity to an increased risk of poor medicine adherence, and two studies on African American adults indicated barriers such as low education and health literacy and poor accessibility to healthcare services [6, 7].

Whilst most people remain in their country of origin, it is estimated that there were 281 million international migrants in the world in 2020 (3.6% of the global population) and 82.4 million people forced to flee their country [8, 9]. Immigrants and refugees are disproportionately affected due to cultural values from their country of origin, language barriers, and migration factors. There is a growing population of immigrants and refugees worldwide; therefore, it is important to be aware of the factors mitigating medicine adherence within this population [10].

There are few studies addressing factors associated with medicine adherence in immigrants and refugees [11–24], and several studies focus on medicine nonadherence in general populations or concentrate on self-care and management of medicines or treatment [25–28]. Although this is the case, current evidence suggests that there is little evidence published that addresses the numerous factors involved in medication nonadherence in immigrants and refugees. Data have not been consolidated and analysed to find common themes for HCPs to target. With increasing concerns regarding the current barriers to adherence in a population inadequately investigated, this study aims to systematically review the rate of adherence and the factors associated with medicine nonadherence in a population of immigrants and refugees.

## 2. Materials and Methods

### 2.1. Search Strategy

A protocol was developed and registered on PROSPERO (CRD42021285419) [29]. Preferred Reporting Items for Systematic Review and Meta-Analyses (PRISMA) guidelines were followed when completing and reporting this systematic review. Electronic databases were searched for literature published between 1st January 2000 and 4th November 2021, and these included PubMed, Medline, Embase, Scopus, CINAHL, and Cochrane Library. A search strategy was conducted based on the following keywords: “immigrant*∗*,” “refugee*∗*,” “medication adherence,” “medicine adherence,” “drug adherence,” “non-adherence,” and “non adherence.” Boolean operators (AND/OR) were used to separate and combine specific key terms, shown in Table 1.

### 2.2. Eligibility Criteria

The PICO framework (population, intervention, comparator, and outcome) was used to formulate the eligibility criteria [30]. Studies investigating factors associated with medicine adherence in immigrants and/or refugees were eligible for inclusion. Studies containing participants <18 years old were excluded as a result of parent/career influence on the patient's medicine adherence.

The terminology used to define medicine adherence has changed over the decades. Compliance was one of the first terms introduced when referring to patients taking their medication as prescribed [31]. This term is no longer used when discussing adherence due to its association with a lack of shared decision-making between the patient and HCP. Thus, studies published prior to 2000 were excluded as more recent studies will use vocabulary that will accurately represent nonadherence. Consequently, fewer synonyms were used when inputting keywords into databases; terms such as compliance, concordance, and treatment persistence were excluded.

The established inclusion and exclusion criteria are shown in Table 2.

### 2.3. Data Extraction and Synthesis

**Figure 1:**
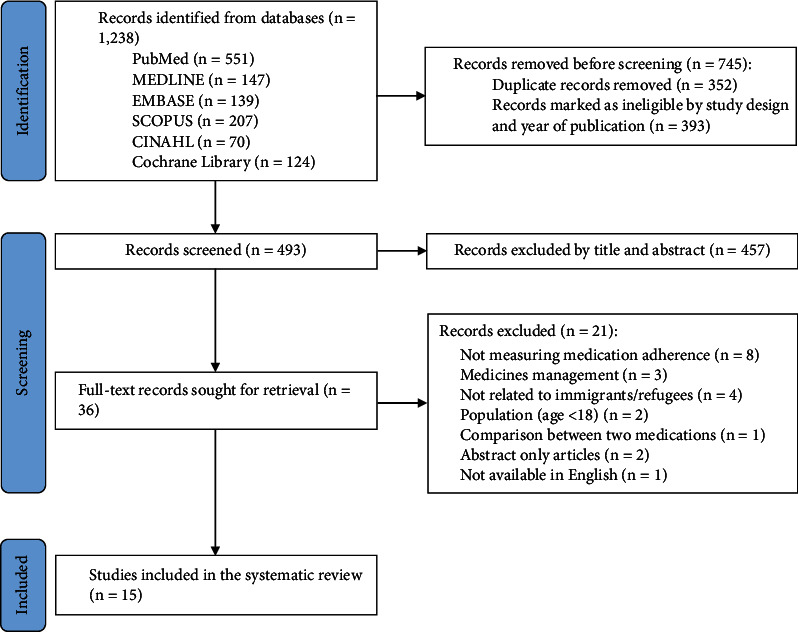
PRISMA flow diagram of the selection process.

Initially, studies were searched in all stated databases using keywords. Prior to exporting the extracted records to a reference management software (EndNote®), records were filtered by date of publication and study design according to the inclusion criteria. After removing duplicate studies, various stages of screening were undertaken for study selection. Titles and abstracts were screened for relevance based on the inclusion criteria. The full text of the remaining studies selected was retrieved and reviewed to determine whether they should be included in this systematic review, and reasons for exclusion were disclosed as shown in the PRISMA flow diagram in Figure 1. All screening against the eligibility criteria was carried out independently by two reviewers (D.P. and Z.J.), and if any disagreements, the third reviewer (P.G.) was consulted.

The data extracted were tabulated using Microsoft Excel© for descriptive and narrative analysis and interpretation. Data extracted included the following: (1) title, (2) author, (3) year, (4) country (study conducted), (5) population studied, (6) study design, (7) condition, (8) number of participants, (9) age, (10) gender, (11) length of study, (12) adherence rates, (13) clinical outcomes, and (14) factors associated with nonadherence. Factors associated with nonadherence were categorised according to the World Health Organisation (WHO) multidimensional adherence model including the five domains: socio-economic factors, healthcare system-related factors, health condition-related factors, therapy-related factors, and patient-related factors [32].

### 2.4. Quality Assessment of Studies

All studies were quality assessed using the National Institute of Health (NIH) quality assessment tool or the Critical Appraisal Skills Programme (CASP) Qualitative Studies Checklist [33, 34]. The NIH tool contains a checklist of fourteen standardised questions suitable for observational, cohort, and cross-sectional studies, and the CASP tool consists of ten questions; they were used to critically appraise each study included in this review. The quality assessment was characterised as good, fair, or poor quality or high, medium, or low quality depending on the assessment tool used.

## 3. Results

### 3.1. Search Results

A total of 1,238 results were identified after searching all electronic databases. After removing duplicates and title and abstract screening, a total of 36 studies remained. Full-text screening of the 36 studies was undertaken to assess eligibility. 15 studies met the inclusion criteria, and the reasons for those excluded are provided in the PRISMA flow diagram in Figure 1.

### 3.2. Quality Assessment Results of Studies Included

All studies included in this review were rated good (score >7) on the NIH Quality Assessment Tool and high (score >7) on the CASP Tool, therefore showing high internal validity. No papers were excluded based on the results of the quality assessment. Most studies assessed using the NIH Quality Assessment Tool lacked internal validity due to the type of study design, and cross-sectional studies measure their exposures and outcomes at the same time rather than separately. Also, the assessors collecting the data were not blinded in any of the studies included in this review. The two papers assessed using the CASP Tool both showed no ethical considerations; however, all other criteria were met [12, 16].

### 3.3. Study Characteristics

A total of 15,322 subjects participated in the 15 selected studies, with studies having a sample size ranging from 25 to 8228 (Table 3). The majority of studies were cross-sectional studies (10 out of 15). Almost half of the studies were conducted in the United States of America (7 out of 15 studies) [11–17]. 3 studies recruited participants from Australia [18–20], 2 studies from Korea [4, 21], and 1 each from the Netherlands [22], Spain [23], and Jordan [24].

Among the studies included, 11 out of 15 exclusively studied immigrant populations. Whilst the remaining 4 studies focused on medicine adherence in refugees, most of the studies (8 out of 15) recruited patients with cardiovascular diseases (CVD), particularly hypertension. 3 studies focused on medicine adherence in patients with human immunodeficiency virus (HIV), 2 studies on antipsychotic adherence for mental health conditions, and 1 study each targeting patients with diabetes and tuberculosis (TB).

### 3.4. Rate of Adherence

Adherence rates were measured in 14 out of 15 studies. The studies used various methods to measure medication adherence, including the following: number of months of completed therapy (1 out of 14), medication possession ratio (MPR) (3 out of 14), self-reported adherence using a Likert scale (1 out of 14), Refills and Medications scale (ARMS) (1 out of 14), Morisky scale (3 out of 14), medication adherence questionnaire (3 out of 14), number of participants never missing a dose (1 out of 14), and number of participants taking their medication as prescribed (1 out of 14).

Studies showed a wide variation of adherence rates, ranging from 10.1% to 74.5% [4, 13]. 6 studies compared the rate of adherence between immigrants and refugees to native or migrant groups [4, 18–21, 23], all of which identified lower adherence rates in the immigrant or refugee population. 4 of the 6 studies showed the results to be statistically significant in comparison to the rate of adherence between immigrants and refugees, and 1 study showed a narrow CI range (95% CI: 1.83–2.21) indicating precise and credible values. Only 1 of the 6 studies showed a wide CI range (95% CI: 25–55% in the native group and 95% CI: 7–31% in the immigrant group (*p*=0.024)), making the results on rate of adherence less reliable.

### 3.5. Clinical and Nonclinical Outcomes

Clinical and nonclinical outcomes of nonadherence to medication were poorly measured. Only 2 out of 15 studies measured the effects of nonadherence on these outcomes. Been et al. stated that nonadherent participants had an increased likelihood of presenting with detectable HIV-RNA; 14.5% in the non-adherent group compared to 6.5% in the adherent group (*P* < 0.05, 95% CI: 1.09–5.48) [22]. Additionally, Forcada et al indicated that patients on antipsychotic treatment that adhered to their medication were less likely to be readmitted to hospital after 12 months, 21.4% compared to 50% in non-adherent patients [23].

### 3.6. Factors Affecting Non-Adherence

The causes of non-adherence are multifactorial, as such the WHO multidimensional adherence model was used to classify these factors into 5 distinctive categories: socio-economic factors, healthcare system-related factors, health condition-related factors, therapy-related factors, and patient-related factors. A breakdown of the factors measured associated with medicine adherence in each study can be found in Table 4.

#### 3.6.1. Socio-Economic Factors

An association between socio-economic factors and medicine non-adherence was found in 14 out of 15 of the studies included. Some of the factors measured in these studies were the number of years of residency, educational attainment, employment status, insurance status, language barriers and social support. 2 studies quantitively measured the effect of employment status on adherence to medication [22, 24]. One of the studies showed a statistically significant correlation to medicine adherence, refugees with no employment had a higher rate of non-adherence (*p*=0.019) due to an inability to pay for their medication [24]. 5 studies investigated the correlation between medicine non-adherence and level of education [11, 18–20, 22]. 3 of those 5 studies, all of which had a similar set of study results, found a weak but significant relationship between higher levels of education in refugees and better medication adherence (*p*=0.003) [18–20].

#### 3.6.2. Healthcare System-Related Factors

Healthcare system-related factors referred to in the studies to communication and relationships between patients and HCPs, accessibility to services as well as continuity of care; this was measured in 5 out of the 15 studies. Baghikar et al. and Vissman et al. found that 7% and 21% of participants, respectively, identified poor communication with their healthcare provider as a barrier to medicine adherence [12, 16]. A further study showed that immigrants that visited an increased number of clinics were associated with a significantly higher likelihood of medicine non-adherence [21]; similar findings were seen by Jang et al. where immigrants who had a constant source of care adhered better to their medication (95% CI: 1.25–2.27) [4]. A study by Abu Khudair et al. conducted in a Syrian refugee camp in Jordan showed a statistical significance result (*p*=0.007) between poor adherence scores and “adequate” explanations from HCPs to patients regarding the causes and complications of hypertension [24].

#### 3.6.3. Health Condition-Related Factors

These factors were recorded in 3 of the 15 studies included. Health condition-related factors included the patient's comorbidities and disease control and characteristics, including severity or lack of symptoms and impact on lifestyle. One study stated that having a comorbidity significantly contributed to an improvement in medication adherence (95% CI: 0.46–0.91) [21]. Another study by Vissman et al. stated that 3% of participants found that suffering from depression was seen as a hindrance to medication adherence [16], comparatively Jang et al. found that immigrants with psychiatric comorbidities had an increased likelihood of medicine adherence [4].

#### 3.6.4. Therapy-Related Factors

Seven out of 15 papers investigated factors such as the side effects of medication, the complexity of the regimen, and the length of therapy. 4 of the 7 studies identified unpleasant adverse effects of medication as an indicator of nonadherence [11, 12, 16, 24], and results ranged from 5.7% to 24% of participants not adhering to their medication because of side effects [11, 16]. Two studies showed that increasing tablet burden improves adherence, a study on antidepressant adherence indicated that taking ≥2 antidepressants contributed to a higher likelihood of adherence (95% CI: 1.67–3.61) [4]. Another study showed that taking ≥2 antihypertensives had a significant association with increased adherence rates (95% CI: 0.55–0.77) [21]. Despite these results, Vissman et al. found that therapy-related factors had no impact on adherence rates in immigrants on highly active antiretroviral therapy (HAART) [17].

#### 3.6.5. Patient-Related Factors

Patient-related factors such as medicine perceptions and beliefs, motivation, health literacy, and lifestyle were linked to medicine nonadherence in immigrants and refugees. These factors were measured in 11 out of the 15 studies included. Been et al. found that immigrants who scored higher stigma scores significantly contributed to nonadherence (*P*=0.001) [22]. Many of the studies concluded that positive medicine beliefs were associated with better medication adherence among immigrant and refugee groups. Attitude and perceived behavioural control towards medicine were statistically significant predictors of medication nonadherence [17]; studies showed immigrants had lower perceived benefits of western medicine [13, 14], and refugees holding “accepting” beliefs than those holding “ambivalent” beliefs had better medication adherence (*p*=0.0001, 95% CI: 0.5–2.4) [18].

## 4. Discussion

This systematic review provides a summary of published papers identifying factors associated with medicine nonadherence and its relationship to the rate of adherence in a population of immigrants and refugees. The results revealed ample data for analysis. The systematic review investigated medicine adherence and its associated factors in immigrants and refugees worldwide suffering from various medical conditions, where the WHO multidimensional adherence model has been used to categorise factors identified. Despite a small sample number of 15 papers, the results among all studies were consistent, with common themes and findings presented. Even though this is the case, not all studies showed clinically significant associations between factors associated with medicine adherence and the rate of adherence.

The results observed aligned with existing literature surrounding this research topic, and all factors measured were associated with medicine nonadherence. Although this was the case, not all studies showed clinically significant results, and not all factors were equally studied; therefore, inferences on the strongest predictors of medicine nonadherence in immigrants and refugees are not possible. Previous studies found that exogenous factors are “more predictive” of rates of adherence than endogenous factors such as age, gender, and ethnicity [35, 36]. Exogenous factors range from patient perceptions of medicine to relationships with HCPs to the adverse effects of medication. This review found that socio-economic factors and patient-related factors were the most studied factors associated with medicine adherence. Medication beliefs and behaviours contributed highly towards medicine nonadherence. In most instances, patients' views are modifiable factors, so theory can be altered. Hence, HCPs should aim to provide targeted advice and clinical interventions to reduce barriers perceived by patients. Studies have also consistently reported economic reasons for medicine nonadherence, and this included a lack of paid employment and an inability to pay for medication. These factors require attention from HCPs and government authorities so that services can be implemented for individuals with low levels of education and those struggling with insurance coverage.

Due to the different methods used to measure the rate of adherence, meta-analysis of the results could not be conducted. The WHO refers to all objective and subjective methods when classifying appropriate methods for assessing medicine adherence [2]. Many of the studies included in this review used self-reported methods to measure the rate of adherence, it is apparent that bias results may be obtained from subjective methods, and using objective methods specific to the type of data being collected could be of benefit to reduce bias [37]. Few studies measured the effect of medication adherence on both clinical and nonclinical outcomes; thus, the extent of the effects of medicine nonadherence cannot be measured. For the papers that did, the results were not clinically significant, and wide confidence intervals were seen. However, previous studies conducted on different populations have shown an association between medicine nonadherence and significant negative clinical and nonclinical outcomes [38, 39].

Medicine nonadherence issues are complex, as such future studies should aim to put an increased emphasis on using objective methods to measure clinical outcomes so that the impact of medicine nonadherence can be quantified; this will also further meta-analysis of data so that trends can be identified using statistic evidence. Currently, this review is broadly focusing on the factors associated with medicine adherence; however, studies should highlight all potential factors linked to nonadherence in a population of immigrants and refugees so that the results are comparable to existing studies. This will allow further statistical analysis of similar factors or themes presented across various studies. It would be good in future studies to have larger sample sizes and be conducted over a longer period of time to allow enough time for medicine taking behaviours to form. Whilst these aspects need to be considered, future research should also identify and investigate interventions to remove common barriers associated with medicine nonadherence in immigrants and refugees. Current studies have touched on the idea; however, evidence on interventions designed for patients in these specific groups could be implemented into everyday practice and used to target and improve individuals' clinical outcomes and reduce health disparities.

### 4.1. Strengths and Limitations

The current review has several limitations and strengths. A high level of heterogeneity was observed in the studies included, and therefore, meta-analysis could not be undertaken; differences included patient characteristics, variations in demographics, and country of origin and study methodologies. The generalisability of the results remains debatable as the outcomes measured were incomparable due to population demographics. The studies included only recruited participants from six countries, of which the majority are developed countries. There are discrepancies between facilities and resources available within different regions and countries, and therefore, the review's findings cannot be generalised to the population worldwide. A further limitation was that most studies included focused on a population of immigrants, and few studies concentrated on factors impacting nonadherence in refugees.

Two quality assessment tools were used based on the study design of the papers included. Despite the outlined limitations, all studies showed good or high internal validity. Six commonly used databases were searched to ensure adequate coverage of the research area being studied. The review provides a comprehensive overview of an area of research that is still emerging. However, further data analysis would have allowed additional correlations between adherence and its associated factors to be made. Nevertheless, the long-term benefits of the findings in this review will provide evidence for the need for interventions among this population.

## 5. Conclusions

This review places importance on the effects of nonadherence, particularly in vulnerable groups like immigrants and refugees, addressing the necessity for further research investigating its effect on clinical outcomes. The results emphasised that socio-economic, patient-related, therapy-related, healthcare system-related, and health condition-related factors are all associated with medicine nonadherence. Notably, socio-economic (including language proficiency, level of education, and financial burden) and patient-related factors (including cultural beliefs and behaviours) contributed highly to nonadherence. Although there is ample literature on medicine adherence, there is limited research focusing on a population of immigrants and refugees. There is scope for future studies to investigate these risk factors quantitatively in more depth, specifically those that are easily modifiable, as well as possible interventions supporting greater adherence in this population for HCPs to implement.

## Figures and Tables

**Table 1:** Search terms.

Electronic database	Search terms
PubMed	((immigrant*∗*) OR (refugee*∗*)) AND ((medication adherence) OR (medicine adherence) OR (drug adherence) OR (non-adherence) OR (non adherence))
Medline	((immigrant*∗* or refugee*∗*) and (medication adherence or medicine adherence or drug adherence or non-adherence or non adherence)).mp
Embase	((immigrant*∗* or refugee*∗*) and (medication adherence or medicine adherence or drug adherence or non-adherence or non adherence)).mp
Scopus	TITLE-ABS-KEY ((immigrant*∗* OR refugee*∗*) AND (medication AND adherence OR medicine AND adherence OR drug AND adherence OR non-adherence OR non AND adherence))
CINAHL	“immigrants OR refugee AND medication adherence OR medicine adherence OR drug adherence OR non-adherence”
Cochrane library	((immigrant*∗* or refugee*∗*) and (medication adherence or medicine adherence or drug adherence or non-adherence or non adherence)).mp

**Table 2:** Eligibility criteria.

Criterion	Inclusion	Exclusion
Population	(i) Immigrants and refugees (ii) Participants >18 years old (iii) Participants presenting with any medical condition requiring medication (iv) No restriction on participants' country of origin	(i) Migrants and asylum seekers (ii) Participants <18 years old

Outcome	(i) Studies reporting factors associated with nonadherence (ii) Studies evaluating rates of adherence	(i) Studies reporting self-management adherence (ii) Studies defining adherence as treatment completion (iii) Studies involving interventions (iv) Studies comparing two medicines

Study design	(i) Cross-sectional studies (ii) Observational studies (iii) Cohort studies (iv) Qualitative studies (v) Mixed method studies	(i) Systematic reviews (ii) Meta-analyses (iii) Case studies/series (iv) Reports (v) Expert opinion articles (vi) Abstracts

Year	(i) Studies published between 1st January 2000 and 4th November 2021	(i) Studies published prior to 2000

Language	(i) Studies in English	(i) Studies in any language other than English

**Table 3:** Study characteristics.

Author	Year	Country (study conducted)	Population studied	Study design	Condition	No. of participants	Age group	Gender	Length of study
Jang et al. [4]	2020	Korea	Immigrants and native-born Koreans who have lived in Korea for more than 6 months (majority living in the city)	Matched cohort study	Mental health	4796	>20	82.8% female, 17.2% male	1 year

Ailinger et al. [11]	2006	United States	Latino immigrants and refugees attending an urban public health clinic in the Washington, DC metropolitan area	Exploratory, cross-sectional	TB	53	18–40, mean age of 27.3 years	64% female, 36% male	9 months

Baghikar et al. [12]	2019	United States	25 Mexican-Americans and 2 Latinos in a low-income, immigrant neighbourhood of Chicago	Qualitative study	Diabetes	27	Mean = 57 years old	81% female, 19% male	6 months

Li et al. [13]	2006	United States	Chinese immigrant from the San Francisco Bay area	Cross-sectional study	CVD	200	>18	50% female, 50% male	1 year

Li and Froelicher [14]	2007	United States	Chinese immigrant adult from the San Francisco Bay area	Cross-sectional study	CVD	200	>18	50% female, 50% male	1 year

Li et al. [15]	2008	United States	Older Chinese immigrants from an Asian health clinic in the San Francisco Bay area	Cross-sectional study	CVD	144	>65	75 men and 69 women	1 year

Vissman et al. [16]	2011	United States	Immigrant Latinos in the south-eastern USA from the Wake Forest University Baptist Medical Centre Infectious Diseases Specialty Clinic (IDSC) and AIDS Care Service (ACS), a local AIDS service organization	Interviews-qualitative study	HIV	25	Range: 25–52	20% female, 80% male	5 months

Vissman et al. [17]	2013	United States	Immigrant Latinos in the south-eastern USA from the Wake Forest Baptist Health	A cross-sectionalclinic-based sample	HIV	66	Range: 23–72	26% female, 74% male	6 months

Shahin et al. [18]	2020	Australia	Middle Eastern refugees and migrants on social pages (e.g., Facebook)	Cross-sectional study	CVD	319	>30	53.7% female, 45.9% male, 0.04% other	10 months

Shahin et al. [19]	2020	Australia	Middle Eastern refugees and migrants recruited from Arabic community groups	Cross-sectional survey	CVD	319	>30	53.7% female, 45.9% male, 0.04% other	10 months

Shahin et al. [20]	2021	Australia	Middle Eastern refugees and migrants from various community groups and English language learning centres	Cross-sectional survey	CVD	319	>18	Refugee: 50.6% female, 49.4% male. Migrant: 57.6% female, 42.4% male	10 months

Cho et. al. [21]	2020	Korea	Immigrants and native-born Koreans (majority living in the city)	Population-based retrospective cohort study	CVD	8228	>30	56.8% female, 33.2% male	3 years

Been et al. [22]	2016	Netherlands	1st and 2nd generation immigrants attending outpatient clinics in Rotterdam	Cross-sectional study	HIV	352	>18	42.6% female, 57.4% male	N/A

Forcada et al. [23]	2013	Spain	Immigrant and native groups from the Acute Psychiatric Unit of the Health Region of Lleida	Retrospective, observational study	Mental health	94	Mean (native) = 35.5, mean (immigrant) = 36.3	Native male = 63.8%, immigrant male = 61.7%	1 year

Abu Khudair et al. [24]	2021	Jordan	Syrian refugees residing in Zaatari camp, Jordan	Cross-sectional study	CVD	180	18–75 years old	53.9% female, 46.1% male	1 month

**Table 4:** Factors measured associated with medicine adherence.

Study	Factors measured associated with medicine adherence				
	Social and economic	Health care system	Health condition	Therapy	Patient
Jang et al. [4]	✔	✔	✔	✔	
Ailinger et al. [11]	✔			✔	✔
Baghikar et al. [12]	✔	✔		✔	✔
Li et al. [13]	✔				✔
Li and Froelicher. [14]	✔				✔
Li et al. [15]	✔				
Vissman et al. [16]	✔	✔	✔	✔	✔
Vissman et al. [17]	✔			✔	✔
Shahin et al. [18]	✔				✔
Shahin et al. [19]	✔				✔
Shahin et al. [20]	✔				✔
Cho et al. [21]	✔	✔	✔	✔	
Been et al. [22]	✔				✔
Forcada et al. [23]					
Abu Khudair et al. [24]	✔	✔		✔	✔
Total	14	5	3	7	11

## Data Availability

The data presented in this study are available upon request from the corresponding author.
